# Risk and protective factors for cognitive decline in Brazilian lower educated older adults: A 15-year follow-up study using group-based trajectory modelling

**DOI:** 10.1016/j.archger.2024.105555

**Published:** 2024-12

**Authors:** Fabiana Ribeiro, Anouk Geraets, Yeda Aparecida de Oliveira Duarte, Anja K. Leist

**Affiliations:** a Department of Social Sciences, University of Luxembourg. Belval Campus,11, Porte des Sciences, L-4366, Esch-sur-Alzette, Luxembourg; b School of Public Health, University of São Paulo – São Paulo, (SP), São Paulo, Brazil

**Keywords:** Cognitive impairment, Aging, Longitudinal studies, Cognitive trajectories, Brazil, Modifiable risk factors, Group-based trajectory modelling

## Abstract

**Background:**

Patterns of cognitive change and modifiable factors for cognitive decline versus stable cognitive trajectories have rarely been described in lower-educated older adults.

**Objectives:**

We aimed to identify long-term trajectories of cognitive functioning and possible factors associated with cognitive decline.

**Design and participants:**

We used data from 1,042 adults aged ≥ 60 participating in the Health, Welfare and Aging Study (SABE), São Paulo, Brazil, without cognitive impairment at baseline. Data were collected across four waves (2000–2015). Group-based trajectory modelling was used to identify cognitive trajectories. Associations with socioeconomic variables, childhood background, lifestyle, and cardiovascular risk factors were explored using weighted multinomial logistic regressions.

**Measurements:**

The abbreviated Mini-Mental State Examination was used to measure cognition.

**Results:**

Three cognitive trajectories were identified: stable (n= 754, 68.6%), mild-decline (n= 183, 20.8%), and strong-decline (n= 105, 10.7%). At baseline, respondents in the strong-decline group were more likely to be older than those with stable and mild-decline trajectories. Furthermore, participants in both the mild and strong-decline groups were more likely to have no schooling, be divorced/separated, receive less than 4 monthly wages, and be underweight (BMI < 18.5) compared to the stable group. Finally, the mild-decline group was more likely to have lived in rural areas during childhood than participants located in a stable trajectory.

**Conclusions:**

Our findings suggest that interventions to reduce cognitive decline for low-educated older adults might include strategies addressing inequalities and improving modifiable risk factor burden.

## Highlights

• Three long-term trajectories of cognitive functioning were identified in a representative sample of older adults in Brazil.
• The group characterised by a stable cognitive trajectory was more likely to identify themselves as white, and report higher education and higher wages.
• Women reached the threshold to cognitive impairment earlier than men.
• Women reported similar disease burden and lower smoking and alcohol consumption prevalence, but lower education compared to men.

## 1 Introduction

Brazil has been experiencing an increase in life expectancy due to improvements in public healthcare access, and consequently a rising number of adults aged 60 years and older, from 14.2 million individuals in 2000 to an estimated 54.2 million individuals in 2040 (Alves, 2016). However, an important point is that although life expectancy has increased from 69.9 years in 2000 to 77.2 years in 2022 (IBGE, 2018), educational levels have not shown similar growth over time among older people in Brazil (Travassos et al., 2020). According to populational data, Brazil has 35% of adults aged 50 or older with primary school level, additionally, 49.0% may be functionally illiterate (Educativa, 2018). It is crucial to understand how these low-educated older adults are ageing, specifically cognitively, as declining cognitive trajectories have been associated with increased healthcare costs, including medical or long-term care costs (Taniguchi et al., 2019).

Studies have shown that higher educational levels are associated with a lower risk for later-life cognitive decline (Rosselli et al., 2022; Zahodne et al., 2015). Educational levels acquired during early life have been argued to contribute up to a 7% reduction in the likelihood of developing dementia (Livingston et al., 2020). The main theoretical framework explaining the effects of higher education level is that of cognitive reserve, which refers to the brain's ability to maintain relatively normal cognitive function despite brain damage and prevent or delay the onset of cognitive decline (Stern, 2002). Indeed, higher education is associated with higher-status professional attainment that contributes to higher cognitive functioning throughout life (Baldivia et al., 2008; Lövdén et al., 2020). Conversely, lower education has been related to unhealthy behaviours, such as reduced physical activity, poor diet, and increased tobacco consumption via lower health literacy (Friis et al., 2016). Furthermore, low education has been proposed as a risk factor for poorer mental health, including higher rates of depression and anxiety (Bjelland et al., 2008). All these factors have been suggested to contribute to an increased risk for dementia (Livingston et al., 2020).

In a recent cross-sectional study examining the association between educational achievement and cognitive performance in older adults from Brazil and Mexico, the findings revealed lower odds of cognitive impairment among Mexican participants compared to their Brazilian counterparts, despite having similar educational levels (Gonçalves et al., 2023). It might be that differences in socioeconomic, lifestyle, and health risk factors between the Brazilian and Mexican older adults contribute to the lower cognitive impairment among the Mexican older adults. Although some cross-sectional studies investigated determinants of cognitive impairment among lower-educated older adults (Brigola et al., 2019; Gonçalves et al., 2023; Suemoto et al., 2022), longitudinal studies that have investigated determinants of cognitive trajectories in homogeneous samples with low educational levels are scarce.

Most studies exploring trajectories of cognitive decline are conducted in high-income countries. Overall, these studies commonly found 3 to 4 heterogeneous trajectories of cognitive decline (Gardeniers et al., 2022; Han et al., 2023; Howrey et al., 2020; Tran et al., 2021, 2021; Wu et al., 2021), in which allocation to moderate and rapid decline was congruently associated with older age and lower education. However, heterogeneity can be observed in these studies, for instance, while in some studies approximately 50% of the study sample had education levels higher than secondary education (Gardeniers et al., 2022; Wu et al., 2021), another study included participants of whom 60.7% had 5 or fewer years of education (Howrey et al., 2020). Moreover, some limitations in these studies can be observed. For example, one study sample was mainly composed of white people (Wu et al., 2021), while another study explored trajectories of a representative Mexican-origin sample of which more than half were born in the U.S. (Howrey et al., 2020). These studies do not allow generalisation to developing countries.

Research coming from developing countries, utilizing representative samples with lower-educated participants from China and employing Mini-Mental State Examination (MMSE) scores to assess cognition, has revealed 2 to 3 different trajectories (Han et al., 2022; Tu et al., 2020). Although both studies utilized MMSE, the variation in the number of identified trajectories can be attributed to the time between measurements and differences in sample composition. Risk factors associated with rapid cognitive decline trajectories included older age, lower education, underweight, unmarried status, and cardiovascular risk factors (Han et al., 2022; Tu et al., 2020).However, several risk factors still need to be investigated, such as childhood background, symptoms of depression, and rurality (Kassouf, 2005). Additionally, it is crucial to recognize that results derived from the Chinese population and may not encompass the unique characteristics present in the Latin American population.

To the best of our knowledge, no studies have employed a group-based trajectory model (GBTM) to examine longitudinal data and assess trajectories of cognitive decline in the Brazilian population using a study sample with homogeneously low levels of education. There is a need to investigate the determinants influencing patterns of cognitive trajectories, within lower-educated populations, as lower education is linked to an increased likelihood of experiencing accelerated cognitive decline or the onset of dementia and major risk factors associated with dementia. GBTM serves as an effective approach to depict the evolution of a specific variable over time and assign it to an appropriate cluster (Nagin & Odgers, 2010). Furthermore, trajectory studies are essential to develop appropriate interventions for cognitively impaired groups, since risk factors can vary among these groups (Nagin, 2010). In this context, the SABE study is the most comprehensive and available longitudinal study performed in one of the most populated capitals of Latin America, including a representative cohort of older adults from São Paulo and being assessed since 2000.

Aiming to extend the previous studies on the topic and to extensively explore the effect of low education by including a more comprehensive set of socioeconomic, and lifestyle/health risk factors, this study aims to provide insight into a) heterogeneous long-term trajectories of cognitive functioning in one of the biggest cities in Latin America, São Paulo (Brazil), b) explore possible sex/gender differences in cognitive decline, and c) investigate socioeconomic, and lifestyle/health factors associated with cognitive decline beyond education, among older adults, that can be modified to delay or reduce cognitive decline.

## 2 Materials and methods

### 2.1 Study population

We used data from the Health, Well-Being, and Aging Study (SABE), an ongoing population-based longitudinal cohort study of older adults aged 60 years and older, living in São Paulo city, Brazil. We utilized available data from assessments that were carried out across four waves (2000, 2005, 2010, and 2015). At the baseline assessment in 2000, a total of 2230 participants were recruited and 2143 participants were interviewed. For this study, we analysed data from 1042 respondents without cognitive impairment at baseline participating in the baseline assessment and at least one follow-up assessment (n = 384 with two assessments, 316 with three, and 342 with four consecutive assessments). Descriptive analyses comparing included and excluded respondents are presented in Appendix Table A1.

**Fig. 1:**
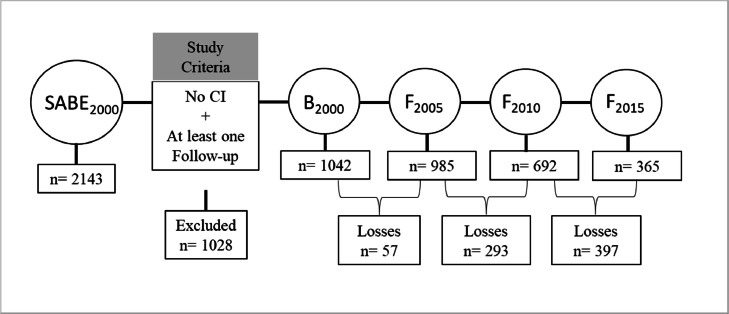
Flowchart of the sample composition of each wave. Note: CI: Cognitive Impairment; B: baseline, F: Follow-up. Although some participants were not located in the second follow-up, they participated in the third or fourth ones.

The SABE study samples were composed of a two-stage stratified sampling design to gather a representative sample as follows: i) The samples were drawn to match the probability sample, and ii) to maintain population representativeness, new cohorts were included at the different waves of the SABE study, for further details see (Lebrão et al., 2018). The SABE study interviews were carried out in person, at participants' homes, by trained interviewers who spoke Portuguese, the official language of Brazil. Additionally, all respondents answered identical questions in the same sequence and phrasing. The sample composition for this study in each wave is provided in Fig. 1.

The SABE was conducted following the Declaration of Helsinki and was approved by The Human Research Ethics Committee at the School of Public Health, University of São Paulo, and the National Committee for Research Ethics. Moreover, in November 2018, the Ethics Review Committee of the European Research Council approved the ethical standards of the Cognitive Aging research project, to which this study is contributing.

### 2.2 Measures

#### 2.2.1 Cognitive functioning – dependent measure

Cognitive functioning was measured by the abbreviated MMSE, which was validated with low-educated adults being less affected by schooling in the Latin American population (Icaza & Albala, 1999; Peláez et al., 2005). This version included a shortened set of questions and tasks that evaluate memory, orientation, attention, language, and visuospatial skills. The maximum score is 19 points and the cut-off point of <= 12 points was used to indicate cognitive impairment, according to validation. In the study, participants were required to complete the MMSE independently, and those who were unable to do so received a score of zero. Consequently, our analysis encompassed both forms of cognitive impairment, with and without dementia.

#### 2.2.2 Baseline characteristics – covariates

Age, sex/gender (men/women), civil status (married/ facto union, single, widowed, and divorced/ separated), self-reported race (white, mixed, black, and others), educational level (no schooling, primary schooling, and more than primary schooling), house ownership (yes/no), number of wages (wage < 1, 1–2 wages, 2–3 wages, 3–4 wages, and wages > 4), rurality (yes/no). Cardiovascular risk factors comprised self-reported responses for hypertension (yes/no), diabetes (yes/no), heart disease (yes/no) and stroke (yes/no). Body mass index was calculated and divided into underweight (<18.5), normal weight (18.5–24.9), overweight (25–29.9), and obese (≥30).

Lifestyle factors comprised alcohol consumption (never, at least 1 time per week, 2–6 times per week, and everyday), smoking (never, former smoking, and currently smoking), depressive symptoms were based on the abbreviated Geriatric Depression Scale (no symptoms, mild symptoms, and severe symptoms) (Almeida & Almeida, 1999), self-reported emptiness (yes/no), physical activity (yes/no), and carrying out artistic activities (yes/no).

Childhood background was assessed by the self-reported economic situation before the age of 15 years (good, regular, and bad), health status before the age of 15 years (excellent, good, and bad), and experiences of hunger/starvation before the age of 15 years (yes/no).

We provide further definitions in the Appendix for some of the baseline measures.

### 2.3 Statistical analyses

We applied group-based trajectory modelling (GBTM) to identify different trajectory groups for cognitive functioning over time. In short, this technique identifies and categorizes individuals with similar longitudinal patterns of a certain outcome. More information about GBTM and the mathematical equation are provided in Appendix.

The MMSE scores were modelled using a censored normal distribution using assessment time as a timescale. This modelling assumes a maximum-probability assignment rule, in which the respondents were assigned to the group corresponding to the highest probability of being members (Nagin & Odgers, 2010). Trajectory models were constructed for 1, 2, and 3 groups, and the shapes of the trajectories were modified by adding higher- or lower-order terms (quadratic or cubic) based on the significance of these terms Model selection is shown in Appendix Table A2. To choose the optimal model, four criteria were utilised: (i) the Bayesian Information Criterion (BIC) with the value closest to zero, which indicates a better fit, (ii) group membership that was statistically significant (p < 0.05), and (iii) entropy, mean posterior probability of membership in each group (values > 0.7 are considered acceptable).

Following the identification of group membership, we performed descriptive statistics that were stratified by trajectory groups. The aim was to depict and contrast the characteristics of the sample obtained from the 2000 baseline wave. Categorical variables were compared by Rao-Scott tests, and continuous variables by adjusted Wald tests. Moreover, cognitive trajectory groups' associations with baseline characteristics were explored by weighted multinomial logistic regression analyses. Before modelling, we assessed for multicollinearity using weighted Pearson's correlation greater than 0.4 as the cut-off (Chan, 2004). The weights and strata design factors from the 2000 SABE survey were applied to all the statistical analyses to produce estimates that were representative of the population.

Finally, we also conducted an analysis stratified by sex/gender, justified by differences between men and women in baseline demographic characteristics. Here, GBTM was run for women and men separately to explore possible trajectory differences.

All analyses were performed using Stata®, version 17 (StataCorp LP, College Station, TX).

## 3 Results

### 3.1 Cognitive functioning trajectories and baseline characteristics

**Fig. 2:**
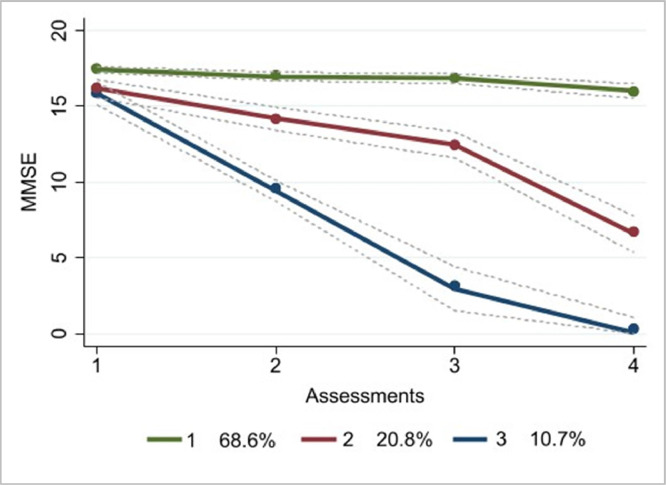
Trajectories of cognitive change among 1042 respondents to the SABE study across 15 years of follow-up; Note: 1 – stable, 2 – mild-decline, 3 – strong-decline.

As shown in Fig. 2, the final GBTM identified three groups based on cognitive performance: “stable” (68.6%), “mild-decline” (20.8%), and “strong-decline” (10.7%) trajectories. The stable trajectory was characterised by a slightly higher starting point on the MMSE and cognitive performance maintenance across time. The strong-decline group was characterised by a slightly lower starting score followed by a rapid decline in the MMSE score from the baseline throughout the follow-ups. The baseline MMSE score of the stable group was significantly higher (*M*= 17.51, *SD*= 1.63) compared to the mild-decline (*M*= 15.95, *SD*= 1.93) and the strong-decline group (*M* = 15.92, *SD*= 1.92), *p* <0.001 (Table 1).

**Table 1:** Baseline sample characteristics by cognitive trajectory group (weighted estimates).

%	Total Sample (n= 1042)	Stable trajectory (n= 754)	Mild-decline trajectory (n= 183)	Strong-decline trajectory (n= 105)	p
Age in years – M (SD)	67.62 (0.36)	66.72 (66.03 - 67.40)	69.75 (68.54 - 70.95)	73.56 (71.96 - 75.16)	<.001^b^
Women	60.65 (57.02 – 64.17)	59.47 (55.28 - 63.53)	68.76 (60.84 - 75.71)	57.06 (46.50 - 67.01)	0.06
*Civil Status*					
Married	61.66 (57.61 - 65.55)	64.13 (59.90 - 68.14)	53.07 (41.48 - 64.34)	51.21 (40.69 - 61.62)	.01*
Single	3.51 (2.32 – 5.28)	3.69 (2.36 – 5.74)	2.16 (0.79 - 5.78)	4.33 (1.50 - 11.80)	
Widowed	26.6 (23.74 - 29.68)	23.77 (20.74 - 27.09)	36.21 (26.78 - 46.83)	39.09 (30.21 - 48.76)	
Divorced/separated	8.23 (6.54 - 10.31)	8.41 (6.52 -10.79)	8.56 (4.68 - 15.14)	5.38 (1.87 - 14.46)	
*Self-identified race*					
White	70.49 (65.53 - 75.75)	73.27 (67.98 - 77.96)	61.72 (51.27 - 71.19)	56.90 (44.89 - 68.15)	<0.001*
Mixed	21.70 (17.59 - 26.46)	18.88 (14.94 - 23.57)	30.04 (20.99 - 40.96)	36.62 (25.07 - 49.95)	
Black	3.10 (2.08 - 4.59)	2.61 (1.58 - 4.28)	4.85 (2.50 - 9.23)	5.05 (2.16 - 11.35)	
Others	4.72 (3.20 - 6.89)	5.24 (3.41 - 7.97)	3.39 (1.28 - 8.68)	1.42 (0.41 - 4.80)	
*Education level*					
No schooling	15.73 (12.42 - 19.73)	10.78 (8.12 - 14.18)	33.95 (23.96 - 45.60)	34.83 (23.50 - 48.17)	
Primary schooling	69.95 (65.27 - 74.24)	71.85 (65.78 - 77.22)	62.89 (51.66 - 72.87)	62.72 (49.42 - 74.33)	< 0.001*
More than primary schooling	14.32 (9.80 - 20.45)	17.36 (11.93 - 24.59)	3.18 (0.82 - 11.42)	2.45 (0.67 - 8.48)	
*House ownership*					
*Yes*	84.18 (80.21 - 87.49)	84.22 (80.03 - 87.67)	84.72 (77.56 - 89.89)	82.6 (72.14 - 89.65)	0.90
*Number of wages*					
Wage < 1	22.46 (18.83 - 26.56)	20.09 (16.17 - 24.69)	31.31 (22.75 - 41.36)	30.23 (21.57 - 40.57)	
1–2 wages	17.10 (14.04 - 20.66)	16.06 (13.01 - 19.67)	20.52 (13.11 - 30.65)	21.35 (12.09 - 34.88)	.002*
2–3 wages	12.68 (10.32 - 15.48)	12.56 (10.05 - 15.60)	12.89 (7.92 - 20.29)	13.47 (7.16 - 23.92)	
3–4 wages	10.49 (8.47 - 12.92)	9.60 (7.60 - 12.06)	13.61 (8.21 - 21.73)	13.76 (6.96 - 25.37)	
wages > 4	37.27 (32.36 - 42.47)	41.68 (36.43 - 47.13)	21.67 (13.98 - 32.0)	21.19 (12.42 - 33.77)	
*Lived in rural areas*					
*Yes*	60.54 (54.67 - 66.13)	57.20 (50.96 - 63.21)	75.04 (65.34 - 82.74)	68.55 (57.68 - 77.70)	< 0.001*
*Cardiovascular risk factors*					
No hypertension	48.46 (44.70 - 52.24)	50.04 (46.06 - 54.02)	45.00 (35.21 - 55.20)	37.42 (26.34 - 50.00)	.14
No diabetes	82.36 (79.07 - 85.23)	83.84 (79.96- 87.10)	76.58 (67.88 - 83.49)	77.48 (67.10 - 85.30)	.07
No heart disease	83.44 (80.90 - 85.69)	83.66 (80.67 - 86.27)	84.02 (76.88 - 89.26)	79.61 (67.81 - 87.85)	.70
No stroke	94.97 (93.09 - 96.36)	96.40 (94.14 - 97.81)	90.33 (83.38 - 94.56)	88.24 (75.28 - 94.87)	.007*
*BMI classes*					
<18.5	1.66 (1.01 - 2.72)	0.68 (0.29 - 1.58)	5.24 (2.59 - 10.30)	5.57 (2.30 - 12.86)	
18.5 – 24.9	32.95 (29.55 - 36.54)	33.69 (30.02 - 37.56)	27.83 (20.54 - 36.52)	35.85 (25.03 - 48.3)3	< 0.001*
25 – 29.9	42.09 (38.59 - 45.66)	42.77 (39.13 - 46.50)	40.90 (31.35 - 51.17)	36.19 (25.99 - 47.81)	
≥ 30	23.31 (20.75 - 26.08)	22.86 (19.78 - 26.26)	26.04 (19.01 - 34.56)	22.39 (14.78 - 32.43)	
*Drinking status*					
Never	13.19 (10.86 - 15.94)	13.95 (11.15 - 17.32)	8.52 (4.84 - 14.57)	14.36 (8.04 - 24.34)	.06
At least 1 time per week	74.41 (70.85 - 77.67)	72.25 (67.85 - 76.25)	85.27 (78.47 - 90.19)	76.35 (64.53 - 85.14)	
2–6 times per week	7.25 (5.07 - 10.26)	8.31 (5.75 - 11.88)	2.92 (1.00 - 8.22)	4.14 (1.27 - 12.63)	
Everyday	5.15 (3.88 - 6.80)	5.49 (4.06 - 7.37)	3.29 (1.38 - 7.62)	5.14 (1.8 0- 13.80)	
*Smoking status*					
Never	56.05 (52.89 - 59.17)	55.42 (51.70 - 59.08)	61.17 (54.34 - 67.59)	52.43 (41.64 - 62.99)	0.42
Former Smoking	13.57 (11.14 - 16.43)	13.59 (10.67 - 17.16)	14.14 (9.86 - 19.87)	12.03 (6.35 - 21.61)	
Currently smoking	30.38 (27.30 - 33.65)	30.98 (27.63 - 34.55)	24.69 (18.87 - 31.61)	35.54 (25.41 - 47.16)	
*Depressive Symptoms*					
No Symptoms	81.67 (79.14 - 83.95)	81.79 (78.92 - 84.34)	80.29 (71.91 - 86.64)	83.22 (73.13 - 90.04)	0.60
Mild Symptoms	14.77 (12.80 - 16.99)	14.69 (12.55 - 17.12)	14.65 (8.76 - 23.50)	15.94 (9.52 - 25.45)	
Severe Symptoms	3.56 (2.36 - 5.35)	3.52 (2.24 - 5.50)	5.05 (2.39 - 10.38)	0.84 (0.12 - 5.83)	
*Self-reported emptiness*					
	27.17 (24.51 - 29.99)	25.66 (22.53 - 29.06)	32.86 (25.49 - 41.18)	32.92 (22.82 - 44.90)	.13
*Physical activity*					
No	67.71 (62.76 - 72.28)	65.10 (59.46 - 70.36)	75.83 (67.73 - 82.43)	80.63 (69.20 - 88.52)	<0.001*
*Manual work, craft, or artistic activity*					
No	65.92 (61.56 - 70.02)	64.87 (60.17 - 69.29)	69.62 (59.84 - 77.90)	70.24 (58.0 4- 80.10)	0.44
*Economic situation before age of 15 years*					
Good	28.67 (25.45 - 32.11)	27.12 (23.49 - 31.08)	37.71 (29.61 - 46.56)	27.32 (18.61 - 38.20)	
Regular	41.59 (37.35 - 46.97)	42.82 (37.79 - 48.00)	35.94 (28.13 - 44.58)	39.44 (28.56 - 51.48)	0.14
Bad	29.74 (26.13 - 33.62)	30.06 (26.07 - 34.37)	26.35 (19.54 - 34.51)	33.24 (23.58 - 44.55)	
*Health before age of 15 years*					
Excellent	49.49 (45.57 - 53.43)	48.62 (44.39 - 52.87)	53.46 (43.33 - 63.31)	51.16 (40.06 - 62.14)	0.59
Good	43.48 (39.81 - 47.22)	44.69 (40.53 - 48.93)	39.42 (30.58 - 49.01)	38.02 (27.73 - 49.51)	
Bad	7.03 (5.49 – 8.97)	6.69 (4.92 - 9.02)	7.13 (3.57 - 13.73)	10.83 (4.56 - 23.56)	
*Starved before age of 15 years*					
No	79.65 (76.29- 82.65)	79.79 (75.88 - 83.21)	79.00 (70.64 - 85.47)	79.42 (69.47 - 86.74)	0.97
MMSE – M(SD)	17.19 (1.76)	17.55 (17.41 – 17.69)	15.87 (15.56 -16.18)	15.87 (15.44 – 16.31)	<.001^b^

Note. M – Mean, SD – standard deviation, MMSE – Mini-Mental State Exam.

a Data are presented as percentages and 95% confidence intervals unless otherwise indicated.

b Weighted *P* value determined using adjusted Wald test or Rao-Scott test (differences among groups were confirmed by non-overlapping of confidence intervals).

⁎ *P* < .05

There were significant differences in the sociodemographic backgrounds among the three groups. As shown in Table 1, participants with a strong-decline trajectory were older compared to those with stable and mild-decline trajectories. Furthermore, strong-decline participants were more likely to self-identify themselves as mixed compared to the stable group. Individuals in both the mild and strong-decline groups reported having no schooling, being divorced/separated, receiving less than 4 monthly wages, and being underweight (BMI < 18.5) compared to individuals in stable trajectory. Finally, the mild-decline group was more likely to have lived in rural areas during childhood than participants located in a stable trajectory.

**Fig. 3:**
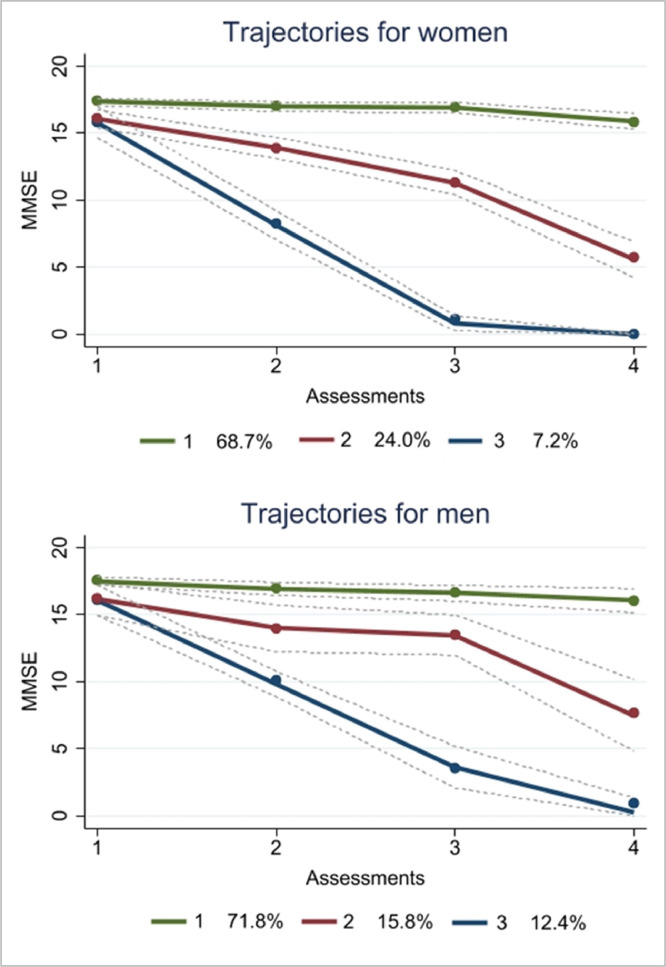
Cognitive functioning trajectories for women (*n*= 648) and men (*n*= 394) across 15 years of follow-up in the SABE study. Note: 1 – stable, 2 – mild-decline, 3 – strong-decline.

Overall, the analyses stratified by sex/gender showed that 71.8% of men were in the stable group, 12% in the strong decline group, and 15.8% were allocated in the mild-decline group, which revealed clinical outcomes for cognitive decline, based on the MMSE cut-off score in the last assessment. In contrast, 68.7% of the women showed stable cognitive functioning, 7.2% showed strong cognitive decline, and 24.0% showed mild-decline, which on average reached the threshold of cognitive impairment in the third assessment (see Fig. 3).

Moreover, descriptive comparisons showed that women were more likely to be widowed, to report receiving less than two wages per month, be obese, have mild and severe symptoms of depression, self-report emptiness, to do manual work, craft, or artistic activity, and consume alcohol one time per week compared to men. In contrast, men were more likely to be current smokers and consume alcohol everyday (see Table 2).

**Table 2:** Baseline characteristics of women versus men (weighted estimates).

%	Women (*n* = 648)	Men (*n* = 394)	p
Age – M (SD)	67.80 (67.03 - 68.58)	67.33 (66.48 - 68.18)	.20
*Civil Status*			
Married	46.76 (42.58 – 50.98)	74.27 (69.61 - 78.43)	< 0.001*
Single	11.24 (8.46 - 14.80)	7.03 (4.57 - 10.65)	
Widowed	39.77 (36.21 - 43.45)	13.76 (10.17 - 18.35)	
Divorced/separated	2.22 (1.18 - 4.13)	4.95 (2.83 - 8.52)	
*Self-identified race*			
White	70.71 (65.86 - 75.13)	70.14 (62.76 - 76.61)	< 0.70
Mixed	20.91 (16.93 - 25.53)	22.91 (16.86 - 30.35)	
Black	3.46 (2.35 - 5.08)	2.54 (1.30 - 4.90)	
Others	4.92 (3.23 - 7.42)	4.40 (2.62 - 7.30)	
*Education level*			
No schooling	18.39 (14.43 - 23.15)	11.66 (8.28 - 16.17)	
Primary schooling	70.94 (66.03 - 75.40)	68.44 (61.39 - 74.73)	< 0.001*
More than primary schooling	10.67 (6.96- 16.02)	19.90 (13.17 - 28.93)	
*House ownership*			
Yes	82.26 (77.90 - 85.92)	87.14 (81.65 - 91.16)	.11
*Number of wages*			
Wage < 1	34.34 (29.45 - 39.58)	8.01 (5.12 - 12.33)	< 0.001*
1–2 wages	21.98 (17.39 - 27.38)	11.16 (8.18 - 15.04)	
2–3 wages	10.55 (7.90 - 13.94)	15.27 (11.23 - 20.44)	
3–4 wages	7.19 (5.43 - 9.44)	14.50 (10.55 - 19.61)	
wages > 4	25.95 (21.71 - 30.69)	51.05 (43.45 - 58.61)	
*Lived in rural areas (yes)*	56.24 (50.75 - 61.58)	67.18 (59.10 - 74.36)	< 0.001*
*Cardiovascular risk factors*			
No hypertension	46.60 (42.43 - 50.82)	51.34 (45.49 - 57.15)	.15
No diabetes	81.17 (77.33 - 84.49)	84.22 (78.52 - 88.62)	.32
No heart disease	84.05 (80.53 - 87.03)	82.49 (78.58 - 85.82)	.53
No stroke	96.12 (94.30 - 97.37)	93.20 (89.69 - 95.57)	.03*
*BMI classes*			
<18.5	1.59 (0.84 - 2.97)	1.77 (0.78 - 3.97)	
18.5 – 24.9	28.47 (24.72 - 32.54)	40.03 (34.071 - 46.29)	< 0.001*
25 – 29.9	38.63 (34.80 - 42.61)	47.55 (42.43 - 52.72)	
≥ 30 (obesity)	31.31 (27.45 - 35.44)	10.66 (7.60 - 14.73)	
*Drinking status*			
Never	12.19 (9.43 - 15.61)	14.75 (11.06 - 19.40)	< 0.001*
At least 1 time per week	83.17 (79.32 - 86.43)	60.90 (53.90 - 67.48)	
2–6 times per week	3.10 (1.93 - 4.94)	13.64 (9.12 - 19.93)	
Everyday	1.54 (0.77 - 3.07)	10.70 (7.89 - 14.37)	
*Smoking status*			
Never	73.63 (69.48 - 77.39)	28.96 (23.68 - 34.88)	< 0.001*
Former Smoking	10.97 (8.46 - 14.10)	17.57 (13.32 - 22.82)	
Currently smoking	15.40 (12.50 - 18.83)	53.47 (47.96- 58.90)	
*Depressive Symptoms*			
No Symptoms	77.84 (74.44 - 80.90)	87.57 (84.23 - 90.28)	< 0.001*
Mild Symptoms	17.59 (14.78 - 20.80)	10.43 (7.68 - 14.01)	
Severe Symptoms	4.57(2.96 - 7.01)	2.01 (0.93 - 4.29)	
**Self-reported emptiness**			
Yes	33.06 (29.47 - 36.87)	17.94 (14.20 - 22.40)	<0.001*
*Physical activity*			
No	70.86 (64.50 - 76.50)	62.8 (55.87 - 69.31)	0.05
*Manual work, craft, or artistic activity*			
No	59.42 (54.46 - 64.20)	75.93 (69.40 - 81.44)	<0.001*
*Economic situation before age of 15 years*			
Good	30.36 (26.27 - 34.80)	26.06 (22.01 - 30.56)	
Regular	42.42 (37.78 - 47.20)	40.32 (34.64 - 46.27)	0.07
Bad	27.21 (23.38- 31.42)	33.63 (28.12 - 39.62)	
*Health before age of 15 years*			
Excellent	47.61 (42.56 - 52.70)	52.40 (45.71 - 59.01)	0.52
Good	45.15 (40.38 - 50.01)	40.90 (35.03 - 47.04)	
Bad	7.24 (5.05 - 10.29)	6.70 (4.27 - 10.35)	
*Starved before age of 15 years*			
No	80.76 (76.58 - 84.35)	77.94 (72.48 - 82.57)	0.36
MMSE – M(SD)	17.10 (16.91 - 17.30)	17.33 (17.13 - 17.53)	.07

Note. M – Mean, SD – standard deviation, MMSE – Mini-Mental State Exam.

a Data are presented as percentages unless otherwise indicated.

^b^ Weighted *P* value determined using adjusted Wald test or Rao-Scott test (differences among groups were confirmed by non-overlapping of confidence intervals).

* *P* < .05

### 3.2 Multinomial logistic regression

Before, carrying out the multinomial logistic regression, we tested the covariates for multicollinearity and we found correlations for self-reported emptiness and depressive symptoms, smoking status and sex/gender, and experience starvation before the age of 15 years and economic situation before 15 years old. However, when we independently removed each one of those variables from the model, we did not observe any differences in the results, suggesting, that multicollinearity was not a significant concern for this model. Testing the potential of baseline characteristics to predict cognitive trajectories (group membership), the following variables were shown to be significantly associated with group membership, p values <0.001: respondents who self-reported their race as mixed, who were older, who had no schooling, and have had a stroke had greater probabilities of being in the mild-decline or strong-decline declining group. Furthermore, respondents with self-reporting hypertension were more likely to be in the strong-decline group compared to the stable group (see Table 3).

**Table 3:** Multinomial logistic regression predicting cognitive trajectory group membership as a function of baseline characteristics.

Predictor in Baseline	Trajectories (Ref = Stable Group)					
	Mild-decline Group			Strong-decline Group		
	**Weighted % (SE)**	**p**	**95% CI**	**Weighted % (SE)**	**p**	**95% CI**
Age	0.08 (0.02)	<0.001*	0.04 - 0.11	0.17 (0.02)	<0.001*	0.12 - 0.21
*Sex (Ref= Women)*						
Men	0.13 (0.32)	0.68	-0.50 - 0.77	- 0.05 (0.43)	0.90	-0.90 - 0.79
*Civil Status (Ref = Single)*						
Married	-0.40 (0.49)	0.42	-1.35 - 0.56	0.22 (0.64)	0.74	-1.03 - 1.46
Widowed	-0.08 (0.46)	0.87	-0.98 - 0.83	0.16 (0 0.64)	0.80	-1.10 - 1.43
Divorced/separated	-0.94 (0.87)	0.28	-2.65 - 0.77	- 19.60 (0.85)	<0.001*	-21.26 - 17.94
*Self-identified race (Ref =White)*						
Mixed	0.72 (0.30)	0.02	0.13 - 1.31	1.12 (0.43)	0.01*	0.27 - 1.96
Black	1.28 (0.54)	0.02	0.22 - 2.35	0.47 (0.74)	0.53	-0.98- 1.92
Others	0.01 (0.69)	0.99	-1.35 - 1.36	-0.64 (0.76)	0.40	-2.13 - 0.86
*Education level (Ref = No Schooling)*						
Primary schooling	-0.80 (0.34)	0.02*	-1.45 - -0.14	-1.18 (0.42)	0.005*	- 2.01- - 0.36
More than primary schooling	-2.01 (0.75)	0.007*	-3.48 - -0.54	-4.30 (1.19)	<0.001*	- 6.62 - -1.96
*House ownership (Ref = No)*						
*yes*	0.09 (0.36)	0.79	-0.60 - 0.79	-0.11 (0.43)	0.81	- 0.96 - 0.74
*Number of wages (Ref=* Wage < 1)						
1–2 wages	0.15 (0.37)	0.68	-0.57 - 0.87	0.60 (0.44)	0.18	- 0.27 - 1.46
2–3 wages	0.20 (0.40)	0.62	-0.59 - 0.99	0.31 (0.64)	0.63	- 0.96 - 1.57
3–4 wages	0.51 (0.46)	0.26	-0.38 - 1.41	0.97 (0.63)	0.12	- 0.26 - 2.20
wages > 4	-0.30 (0.41)	0.46	-1.10 - 0.50	0.15 (0.50)	0.76	- 0.82 - 1.13
*Lived in urban areas (Ref = Yes)*						
*No*	-0.30 (0.30)	0.32	-0.89 - 0.30	-0.10 (0.39)	0.80	- 0.87 - 0.67
*Cardiovascular risk factors*						
Hypertension (*Ref =* No)						
Yes	-0.18 (0.28)	0.50	-0.73 - 0.36	0.66 (0.33)	0.04*	- 0.21 – 1.65
Diabetes (*Ref =* No)						
Yes	0.59 (0.33)	0.07	-0.06 - 1.24	0.80 (0.43)	0.06	-0.05 - 1.65
Heart disease (*Ref =* No)						
Yes	-0.17 (0.35)	0.63	-0.88 - 0.52	-0.24 (0.45)	0.59	-1.12 - 0.64
Stroke (*Ref =* No)						
Yes	1.72 (0.58)	0.003*	0.57 - 2.86	2.01 (0.72)	0.005*	0.61 - 3.42
*BMI class (Ref = <18.5)*						
18.5–24.9	-0.95 (0.82)	0.25	-2.57 - 0.66	-1.30 (0.95)	0.17	- 3.16 - 0.60
25–29.9	-0.82 (0.82)	0.32	-2.43 - 0.79	-1.43 (0.96)	0.14	- 3.32 - 0.45
≥ 30	-0.96 (0.84)	0.25	-2.61 - 0.68	-1.53 (0.97)	0.11	- 3.43 - 0.37
*Drinking status (Ref = Never)*						
At least 1 time per week	0.52 (0.43)	0.22	-0.32 - 1.36	-0.39 (0.45)	0.38	- 1.27 - 0.49
2–6 times per week	0.16 (0.68)	0.81	-1.16 - 1.50	-0.21 (0.77)	0.78	- 1.72 - 1.30
Everyday	0.25 (0.82)	0.76	-1.37 - 1.86	-0.49 (0.98)	0.62	- 2.41 - 1.42
*Smoking status (Ref =*Currently smoking*)*						
Former Smoking	0.65 (0.45)	0.15	-0.24 - 1.53	0.78 (0.59)	0.18	- 0.37 - 1.94
Never	0.20 (0.31)	0.52	-0.41 - 0.81	0.19 (0.44)	0.66	- 0.67 – 1.04
*Depression Symptoms (Ref =No depression)*						
Mild Depression	-0.55 (0.45)	0.22	-1.43 - 0.34	-0.10 (0.56)	0.86	- 1.20 - 1.00
Severe Depression	0.37 (0.97)	0.71	-1.55 - 2.28	-0.40 (1.19)	0.73	- 2.73 – 1.93
Self-reported emptiness *(Ref = yes)*						
No	-0.14 (0.33)	0.68	-0.79 - 0.52	0.02 (0.41)	0.97	- 0.78–0.82
*Physical activity (Ref = no)*						
Yes	0.05 (0.30)	0.88	-0.54 - 0.63	-0.01 (0.43)	0.98	-0.86 - 0.84
*Manual work, craft, or artistic activity*						
Yes	-0.05 (0.28)	0.86	-0.59 - 0.49	0.16 (0.41)	0.69	-0.64 - 0.96
*Economic situation before the age of 15 years (Ref = Regular)*						
Good	0.22 (0.30)	0.47	-0.37 - 0.80	0.18 (0.39)	0.64	-0.58 - 0.95
Bad	-0.31 (0.35)	0.36	-0.99 - 0.36	0.36 (0.50)	0.47	- 0.62 - 1.33
*Health before the age of 15 years (Ref= Good)*						
Excellent	0.27 (0.28)	0.34	-0.28 - 0.82	0.30 (0.33)	0.33	-0.34 - 0.94
Bad	0.66 (0.50)	0.18	-0.31 - 1.63	-0.09 (0.76)	0.90	-1.59 - 1.40
*Starved before the age of 15 years (Ref = no)*						
Yes	-0.08 (0.37)	0.82	-0.80 - 0.64	-0.60 (0.59)	0.31	-1.75 - 0.55

Legend: Ref: Reference variable, SE: Standard error, CI: confidence interval, BMI: Body Mass Index

*P < .05

## 4 Discussion

### 4.1 Summary and explanation of findings

To our knowledge, this study is the first to investigate long-term trajectories of cognitive functioning in a representative sample of lower-educated older adults in Brazil, in addition to explore the role of diverse modifiable socioeconomic, lifestyle, and cardiometabolic factors in these trajectories. We identified three trajectories of cognitive functioning using GBTM, which extends previous studies (Howrey et al., 2020; Tran et al., 2021; Tu et al., 2020) with shorter and less frequent follow-up duration. This finding is in contrast with earlier research identifying more than three cognitive trajectories in highly educated older adults performed in developed countries (Gardeniers et al., 2022; Wu et al., 2021). Aside from differences in the study population, these studies included annual assessments over a shorter follow-up duration (Wu et al., 2021), which might have captured more noise and did not inform about long-term differences. Further, results could to some extent depend on the sample composition as the process of cognitive ageing is not homogeneous but rather heterogeneous (Han et al., 2022).

Most participants in our sample had a higher likelihood of being allocated in the stable trajectory (68.6%) across the measurement years, while the probability of being in the strong-decline group membership was 10.7%. According to the MMSE classification, the mild-decline group reached the average threshold for cognitive impairment on the last follow-up, while the strong-decline group reached the threshold for cognitive impairment on the second follow-up. Moreover, both mild- and strong-decline participants presented higher average age and lower MMSE scores at baseline compared to the stable trajectory. These findings hold significant implications for diagnosing and implementing customised treatment strategies aimed at delaying cognitive decline in this at-risk population of older adults.

Regarding gender-stratified trajectories, we observed a higher percentage of men being allocated in the strong-decline trajectory, while more women were in the mild-decline group. Furthermore, women seem to have a threshold for cognitive impairment in the third assessment, while men reached this threshold in the last assessment. These results could rely on the fact that women in Brazil have historically faced educational barriers, including less access to formal education leading to lower incomes and less access to financial resources (Ribeiro et al., 2023). Consequently, this would limit their access to adequate health care, including early detection and treatment of cognitive impairment. Indeed, in our data women were more likely to be lower educated and receive less than one wage per month.

When comparing the demographic characteristics of trajectory groups, we observed differences in factors classically found in the literature, such as education, but also in factors related to low education in a Brazilian-specific social environment, such as race and lower wages. Lower education among older people in Brazil is related to the fact that public education quality is low (Gonçalves et al., 2023). And this is extremely relevant, since educational levels are also related to prospects of the type of job and, consequently, the type of work developed, in other words, those with higher education levels, have complex or intellectually demanding jobs leading to cognitive reserve, which in addition associates with higher income (Baldivia et al., 2008).

Another factor that was observed in the mild-decline group was a higher likelihood of having lived in rural areas during their childhood. In the Brazilian context, it is important to mention that medical assistance only covers 40% of the attempts to access public health assistance in rural regions. Furthermore, almost 50% of individuals attempting to seek care abandon their efforts due to extended waiting times (Kassouf, 2005). Residing in countryside regions may also constrain individuals to pursue jobs that are less mentally challenging, such as agricultural work, potentially resulting in reduced cognitive abilities in later life (Saenz et al., 2018).

Additionally, we also identified that divorced/separated respondents were more likely to be allocated in the mild and strong-decline groups, one possible explanation for this result is that people in this civil status might experience greater isolation (Cornwell, 2012) leading to lower cognitive engagement (Jennings et al., 2022). Another point to highlight is the fact that this result seems to be more common in countries where separation or divorce is seen as a stigma in society (Jennings et al., 2022), which is the case in Brazil.

Regarding the multinomial logistic regression, though several differences were found in terms of socioeconomic, lifestyle, and health factors among groups, only race, education, and being divorced/separated were associated with trajectories in cognitive functioning. Indeed, both race and education have been linked to later-life cognitive decline (Quiñones et al., 2022). Previous studies suggested that race and socioeconomic factors cannot be separated (Zeng et al., 2022). Furthermore, life course risks on cognition among mixed and black participants might suggest the existence of other underlying mechanisms. These mechanisms could include experiences of discrimination, neighbourhood effects, or structural barriers, such as limited access to educational or healthcare resources (Phelan & Link, 2015). Regarding marital status, as shown by Jennings et al., (2020) participants who are divorced/separated might have lower emotional support than married participants, which can lead to poorer cognitive functioning.

### 4.2 Strengths and limitations

Our study has several advantages, we investigated a wide range of modifiable factors that were previously indicated as possible predictors of cognitive decline. Additionally, our study included a sample of individuals with low educational attainment, in which 16% of the sample had no education, a characteristic commonly found in developing and low-income countries. Finally, GBTM can identify distinct trajectories of cognitive decline, which can provide a more nuanced understanding of the heterogeneity of cognitive ageing. Furthermore, subsequent analyses carried out comparing trajectories can inform the development of targeted interventions to prevent or delay cognitive decline. Identifying the risk factors for cognition, beyond education, such as hypertension, and stroke could aid in developing preventive strategies to tackle the challenging issue of rising dementia rates worldwide, particularly among older adults in low- and middle-income countries (Tu et al., 2020). Finally, despite experiencing participant losses over the years due to reasons such as death or dropout, it is crucial to acknowledge that in the context of random sample losses, GBTM utilizing maximum likelihood estimates offer parameter estimates that are asymptotic unbiasedness (Nagin & Odgers, 2010).

The study comes with some limitations; the MMSE measure suffers from ceiling effects and insufficient sensitivity in identifying subtle cognitive impairment (Franco-Marina et al., 2010). Moreover, although MMSE is able to identify participants’ memory issues, difficulties in learning new information, concentrating, and/or making decisions, and hindering regular daily activities, this measure alone is not able to distinguish individuals with mild cognitive impairment (Petersen, 2004) or dementia (Jack et al., 2011), which would be important for checking specific risk factors for these conditions. Another point to highlight is the inclusion of only those without cognitive impairment and with at least one follow-up from 2000 (baseline) which may have resulted in a healthier study sample with less variation in cognitive function and an underestimation of cognitive impairment, as shown by the comparison between excluded and included samples. Furthermore, the time between assessments was five years and it is not possible to capture different nuances of cognitive decline changes.

Lastly, we were not able to carry out multivariate analysis stratifying by group and sex, because of the small number of participants in the strong-decline group. Due to the limitations mentioned, future studies should include shorter time assessments and also a larger sample size to enable the performance of stratified analyses, thereby increasing statistical power. Overall, the study suggests that risk factors for cognitive decline in this sample of lower-educated older adults are similar to those found in other mainly white, wealthier, and higher-educated populations, such as older age, lower educational attainment, and the presence of chronic diseases. In this context, the development and implementation of robust public policies is crucial. These policies should focus on identifying individuals at risk for cognitive decline, instituting regular monitoring, and implementation of targeted interventions to prevent or delay cognitive decline.

## Funding

This work was supported by the European Research Council (ERC) under the European Union's Horizon 2020 research and innovation programme [Grant number No. 803239 to AKL].

## CRediT authorship contribution statement

**Fabiana Ribeiro:** Conceptualization, Methodology, Software, Formal analysis, Writing – original draft. **Anouk Geraets:** Visualization, Writing – review & editing. **Yeda Aparecida de Oliveira Duarte:** Investigation, Data curation. **Anja K. Leist:** Funding acquisition, Writing – review & editing, Supervision.

## Declaration of competing interest

None.

## Data Availability

The current study's datasets are not accessible to the public because of ethical limitations. However, interested parties can obtain these datasets by making a reasonable request to the corresponding author.

## Appendix Supplementary materials

Image, application 1
