# Supplementary material for: Risk and protective factors for cognitive decline in Brazilian lower educated older adults: A 15-year follow-up study using group-based trajectory modelling
Source: Arch Gerontol Geriatr. 2024 Dec;127:None. doi: 10.1016/j.archger.2024.105555 (PMC11413524; doi:10.1016/j.archger.2024.105555)
Supplement: Supplementary file 1 [file mmc1.docx]

**Supplementary Material**

**Table A1.**

Baseline characteristics of included and excluded respondents (weighted estimates).

| % | **Total Sample**  (n= 2151) | **Included**  (n= 1042) | **Excluded**  (n= 1109) | **p** |
| --- | --- | --- | --- | --- |
| Age – M (SD) | 67.33 (5.88) | 67.62  (66.90 – 68.34) | 71.45  (70.41 – 72.48) | <.001* |
| Women | 1267 (58.90%) | 60.7 (57.0 – 64.2) | 55.9 (52.1 – 59.7) | 0.07 |
| *Civil Status* |  |  |  |  |
| Married | 1002 (48.95%) | 57.7 (54.0 – 61.3) | 48.7 (43.8 - 53.7) | < 0.001* |
| Single | 182 (8.89) | 9.6 (7.6 – 12.0) | 12.2 (9.5 - 15.6) |  |
| Widowed | 822 (40.16) | 29.4 (26.2 – 32.8) | 37.5 (33.2 - 41.9) |  |
| Divorced / separated | 41 (2) | 3.3 (2.1 – 5-1) | 1.6 (0.9 - 2.8) |  |
| *Self-identified race* | | | | |
| White | 529 (71.18) | 70.5 (65.5 - 75.0) | 69.5 (63.7 - 74.8) | <.39 |
| Mixed | 422 (19.65) | 21.7 (17.6 - 26.5) | 20.5 (16.4 - 25.4) |  |
| Black | 87 (4.05) | 3.1 (2.1 - 4.6) | 4.9 (3.2 - 7.7) |  |
| Others | 110 (5.12) | 4.7 (3.2 - 6.9) | 5.0 (3.4 - 7.3) |  |
| *Education level* |  |  |  |  |
| No schooling | 524 (24.58%) | 15.7 (12.4 - 19.7) | 27.5 (22.5 – 33.2) |  |
| Primary schooling | 1361 (63.84) | 69.9 (65.3 - 74.2) | 59.0 (53.4 – 64.5) | < .001* |
| More than primary schooling | 247 (11.59) | 14.3 (9.8 – 20.5) | 13.4 (9.4 – 18.7) |  |
| *House ownership (Yes)* | | | | |
|  | 1703 (79.28) | 84.2 (80.2,87.5) | 72.0 (67 – 76.4) | < .001* |
| *Number of wages* | | | | |
| Wage < 1 | 544 (29.79) | 22.5 (18.8 - 26.6) | 32.0 (27.6 - 36.6) | < .001* |
| 1-2 wages | 346 (18.95) | 17.1 (14.0 - 20.7) | 15.9 (12.8 - 19.5) |  |
| 2-3 wages | 242 (13.25) | 12.7 (10.3 - 15.5) | 13.4 (10.8 - 16.6) |  |
| 3-4 wages | 191 (10.46) | 10.5 (8.5 - 12.9) | 12.3 (9.6 - 15.5) |  |
| wages > 4 | 503 (27.55) | 37.3 (32.4 - 42.5) | 26.5 (22.3 - 31.2) |  |
| *Lived in rural areas* |  |  |  |  |
|  | 1376 (64.09) | 60.5 (54.7 - 66.1) | 65.2 (58.3 - 71.6) | 0.17 |
| *Cardiovascular risk factors* | | | | |
| No hypertension | 984 (46.07) | 48.5 (44.7 - 52.2) | 44.2 (40.8 - 47.7) | .07 |
| No diabetes | 1750 (82.24) | 82.4 (79.1 - 85.2) | 81.7 (78.1 - 84.9) | .80 |
| No heart disease | 1681 (78.59) | 83.4 (80.9 - 85.7) | 76.7 (73.3 – 79.8) | < .001* |
| No stroke | 1976 (92.08) | 95.0 (93.1 - 96.4) | 90.2 (88.4 - 91.7) | < .001* |
| *BMI classes* | | | | |
| <18.5 | 69 (3.82) | 1.7 (1.0 - 2.7) | 5.1 (3.6 - 7.2) |  |
| 18.5 – 24.9 | 673 (37.31) | 32.9 (29.6 - 36.5) | 40.4 (36.1 - 44.8) | < .001* |
| 25 – 29.9 | 694 (38.47) | 42.1 (38.6 - 45.7) | 35.7 (32.1 - 39.5) |  |
| ≥ 30 | 368 (20.40) | 23.3 (20.7 - 26.1) | 18.8 (15.8 - 22.1) |  |
| *Drinking status* | | | | |
| Never | 240 (11.16) | 13.2 (10.9 - 15.9) | 9.4 (7.8 - 11.4) | .01* |
| At least 1 time per week | 1661 (77.22) | 74.4 (70.9 - 77.7) | 77.5 (74.6 - 80.2) |  |
| 2-6 times per week | 126 (5.86) | 7.2 (5.1 - 10.3) | 5 (3.6 - 6.8) |  |
| Everyday | 124 (5.76) | 5.1 (3.9 - 6.8) | 8.1 (6.2 - 10.4) |  |
| *Smoking status* |  |  |  |  |
| Never | 1157 (53.81) | 56.1 (52.9 - 59.2) | 47.2 (43.8 - 50.6) | < .001* |
| Former Smoking | 294 (13.67) | 13.6 (11.1 - 16.4) | 18.7 (15.8 - 22.0) |  |
| Currently smoking | 699 (32.51) | 30.4 (27.3 - 33.7) | 34.1 (30.5 - 37.8) |  |
| *Depressive Symptoms* | | | | |
| No *Symptoms* | 1529 (81.63) | 81.7 (79.1 - 83.9) | 79.8 (76.5 - 82.8) | 0.62 |
| Mild *Symptoms* | 276 (14.74) | 14.8 (12.8 - 17.0) | 16.7 (14.0 - 19.9) |  |
| Severe *Symptoms* | 68 (3.63) | 3.6 (2.4 - 5.4) | 3.5 (2.4 - 5.0) |  |
| Self-reported emptyness | 569 (31.03) | 27.2 (24.5 - 30.0) | 32.9 (29.5 – 36.5) | <.001* |
| *Physical activity* | | | | |
| No | 1658 (77.12) | 67.7 (62.8 - 72.3) | 79.9 (75.6 – 83.6) | <.001* |
| *Artistic activities* | | | | |
| No | 1471 (68.48) | 65.9 (61.6 - 70.0) | 73.2 (69.2 - 76.8) | 0.003* |
| *Economic situation before age of 15 years* | | | | |
| Good | 847 (39.95) | 41.6 (37.3 - 46.0) | 37.4 (34.1 - 40.8) |  |
| Regular | 660 (31.13) | 28.7 (25.5 - 32.1) | 31.9 (28.0 - 36.1) | 0.18 |
| Bad | 613 (28.92) | 29.7 (26.1 - 33.6) | 30.7 (26.9 - 34.7) |  |
| *Health before age of 15 years* | | | | |
| Excellent | 973 (45.72) | 43.5 (39.8 - 47.2) | 46.4 (42.2 - 50.6) | 0.06* |
| Good | 1031 (48.45) | 49.5 (45.6 - 53.4) | 49.1 (44.5 - 53.7) |  |
| Bad | 124 (5.83) | 7.0 (5.5 - 9.0) | 4.6 (3.4 - 6.2) |  |
| *Starved before age of 15 years* | | | | |
| No | 1716 (81.29) | 79.7 (76.3 - 82.6) | 80.7 (77.9 - 83.3) | 0.47 |
| MMSE – M(SD) | 17.33 (1.67) | 17.51 (17.04 – 17.35) | 15.56 (15.24 – 15.88) | <.001* |

Note. M – Mean, SD – standard deviation, MMSE – Mini Mental State Exam.

a Data are presented as percentages unless otherwise indicated

b Weighted *P* value determined using adjusted Wald test or Rao-Scott test.

* *P* < .05

*Baseline characteristics – Covariates*

Participants' self-reported race was categorized based on their response to a list of options that align with Brazil's official skin colour classification (Silva, 1999). The question asked was "Which of these options best describes you?" and the available options were white, brown or mixed, black, yellow, and indigenous. As there were relatively few cases in the yellow and indigenous categories, participants who identified as such were grouped under the category 'other'.

Individual income was determined based on the national minimum wage (NMW) in the year of the interview. The NMW for each year was: R$151 in 2000, R$350 in 2006, R$510 in 2010, and R$788-R$880 in 2015/2016. To standardize the data, the Brazilian real (R$) income was recalculated based on the number of times the NMW would need to be multiplied to reach the same amount. This resulted in income categories ranging from 1-2 times the NMW to over 4 times the NMW.

Body weight was measured objectively using height determined by applying a stadiometer objectively measured using a calibrated scale. Body mass index (BMI) was calculated using each participant's weight in kilograms (W) and height in meters (h), with the formula: BMI = W/h2. Based on their calculated BMI, participants were classified into one of four categories: underweight (<18.5), normal weight (18.5 – 24.9), overweight (25 – 29.9), or obese (≥30).

Self-reported cardiovascular risk factors were assessed by the following questions for each of the variables (heart disease, i.e., congestive heart failure, coronary heart disease or occurrence of a heart attack, stroke, diabetes, hypertension): “*Has a doctor or nurse ever told you that you had....?”*

Regarding rurality, participants replied (Yes or No) to the following question: *Until the age of 15, Did you live in the countryside for 5 years or more?*

Drinking Status was based on the responses to the question: *In the last 3 months, on average, how many days a week did you drink alcohol?* And Smoking status was based on the following question: *Do you have or had the habit of smoking?*

Regarding self-reported emptiness, respondents answered the following question with yes or no: Do you feel that your life is empty?

The abbreviated Geriatric Depression Scale (Almeida & Almeida, 1999) was utilized to evaluate depressive symptoms, with the number of symptoms serving as the basis for classifying them as either none, mild, or severe. Mild depression is defined as scoring between 6 to 10 points, while severe depression is characterized by scoring 11 points or higher.

**For physical activity** - In the last 12 months, have you exercised or performed vigorous physical activity regularly, such as sports, brisk walking, dancing, or heavy lifting, 3 times a week?

**Self-reported artistic activities -** In the last 12 months, did you regularly do some manual work, craft or artistic activity, at least once a week, to entertain yourself?

Reference

Almeida, O. P., & Almeida, S. A. (1999). Confiabilidade da versão brasileira da Escala de Depressão em Geriatria (GDS) versão reduzida. )Reliability of the Brazilian version of the Geriatric Depression Scale (GDS) short form) *Arquivos de Neuro-Psiquiatria*, *57*(2B), 421–426. https://doi.org/10.1590/S0004-282X1999000300013

Silva, N. V. (1999). Morenidade: Modos de usar. Cor e estratificação social. (Morenity: Ways of wearing. Color and social stratification.) In *Cor e estratificação social* (pp. 86–106). Contracapa.

**Group-based Trajectory Modelling Equation.**

Group-based trajectory modelling (GBTM) was developed to study patterns of criminal behaviour. However, more recently this technique has been used in epidemiological and clinical research to investigate patterns of health related outcomes(Jones & Nagin, 2013).

In the context of GBTM, the key focus revolves around the distribution of outcomes conditional on assessments; that is, the distribution of outcome trajectories denoted by $P{(Y}_{i}{|Assessments}_{i})$ , where in our study the random vector $Y_{i}$ represents longitudinal sequence of MMSE scores to evaluate cognitive functioning and the vector ${Assessments}_{i}$ represents the SABE year of assessment. The group-based trajectory model posits that the distribution of trajectories within the population is derived from a finite mixture of unknown order J. The likelihood for each respondent MMSE scores i, conditional on the number of groups *J*, written below.

$$P{(Y}_{i}{|Assessments}_{i})=\sum_{j=1}^{J} \pi^{j} \times P\left( Y_{i}|{Assessments}_{i},j;\beta^{j} \right),$$

$\pi^{j}$ represents the probability of belonging to group j, and the conditional distribution of $Y_{i}$ given membership in j is characterized by the unknown parameter vector $\beta^{j}.$ This vector, among other factors, influences the configuration of the group-specific trajectory. Consequently, in GBTM, individuals with analogous longitudinal patterns of outcomes are recognized and categorized together. GBTM assumes the existence of unrecognized subpopulations or distinct groups based on their trajectories over time.

**Reference**

Jones, B. L., & Nagin, D. S. (2013). A Note on a Stata Plugin for Estimating Group-based Trajectory Models. *Sociological Methods & Research*, *42*(4), 608–613. https://doi.org/10.1177/0049124113503141

**Table A2.**

Model selection of cognitive functioning trajectories.

| **Number of classes/polynomial order** | **Bayesian information criterion** | **Entropy** |
| --- | --- | --- |
| MMSE |  |  |
| 1 / 3 | -7553.98 | / |
| 2 / 2 3 | -7119.35 | 0.82 |
| 3 / 2 3 3* | **-7055.23** | **0.73** |
| 4 / 2 2 3 3 | -7014.93 | 0.59 |
| 5 / 3 2 3 3 3 | -6943.83 | 0.53 |

* The model selected. A BIC closer to 0 indicates a better fit. Entropy values > 0.7 are considered acceptable.
